# Real‐world dose adjustments of biologic treatments in psoriasis and their economic impact: a Swedish national population study

**DOI:** 10.1111/ced.15288

**Published:** 2022-09-06

**Authors:** Alexander Egeberg, Jonatan Freilich, M. Natalia Stelmaszuk, Rikke Kongerslev, Eydna Apol, Jes Birger Hansen, Lars‐Åke Levin

**Affiliations:** ^1^ Department of Dermatology Bispebjerg Hospital, University of Copenhagen Copenhagen Denmark; ^2^ Department of Public Health and Clinical Medicine Umeå University Umeå Sweden; ^3^ Parexel International Stockholm Sweden; ^4^ LEO Pharma A/S Ballerup Denmark; ^5^ Department of Health, Medicine and Caring Sciences (HMV) Linköping University Linköping Sweden

## Abstract

**Background:**

To date, evidence on the dose adjustments of biologics in the real‐world treatment of psoriasis is limited. However, dose adjustments may have important clinical and economic implications.

**Aims:**

To study the dose adjustments of individual biologics over time in real‐world practice in Sweden.

**Methods:**

A retrospective observational study of adults with moderate to severe psoriasis was conducted based on Swedish national registry data from 2010 to 2018. Treatment episodes were identified for individual patients from the date of drug dispensation to the end of the supply of the drug. Dosing data were expressed as the proportion of treatment episodes with accumulated syringes/vials equal to, above or below the recommended guidelines. Real‐world costs were calculated and compared with costs predicted from dosing guidelines.

**Results:**

The mean dose was above recommended levels for all biologics investigated. Weighted mean dose adjustments for adalimumab, etanercept, secukinumab and ustekinumab were 13%, 23%, 8% and 3%, respectively, over the entire treatment period. Higher doses translate to higher costs, including notable increases over time vs. expected costs for secukinumab.

**Conclusions:**

Dose adjustments of biologics are frequent in clinical practice but differ for the various biologics. The mean observed increases in dose above guideline recommendations might indicate perceptions of suboptimal efficacy for biologics, with implications for the cost and cost‐effectiveness of these treatments. Further research is warranted to understand the reasons for dose adjustments in clinical practice.

## Graphical abstract

Dose adjustments of biologics are frequent in clinical practice, but few studies have analysed the dose adjustments of individual biologics in the real‐world treatment of moderate to severe psoriasis. This patient‐level registry study from Sweden shows that the mean dosing of biologics is above guideline recommendations, with a trend to increased dosing over time. This increase in dosing might indicate that current biologic therapies are frequently perceived to be suboptimal.

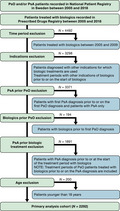

## Introduction

Insights into the pathogenesis of psoriasis have led to the development of targeted biologic therapies capable of modifying the immune response.^1^ Biologics are more effective than nonbiologic treatments, while newer biologics that directly target interleukin (IL)‐17 or IL‐23 show enhanced efficacy in phase III trials compared with older biologics targeting tumour necrosis factor (TNF)‐α^2^, ^3^, ^4^, ^5^ or IL‐12/23.^3^, ^6^, ^7^

There are limited data to confirm how the characteristics of biologics demonstrated in clinical trials translate into clinical practice,^8^ and this is particularly evident for newer biologics. Real‐world experience suggests that efficacy is reduced in many patients at doses established in clinical trials, which may result in worsening symptoms, exacerbation of comorbidities and reduced quality of life.^9^, ^10^, ^11^, ^12^, ^13^ Consequently, doses of biologics may be increased above guideline recommendations, dosing frequency may be increased, or patients may be switched to alternative biologics with the expectation of improved efficacy or tolerability.^11^, ^14^, ^15^, ^16^, ^17^, ^18^ Alternatively, in patients who achieve remission, there may be a reduction in dose to reduce the risk of adverse events (AEs).^19^

Biologic therapies are costly and dose adjustments have economic implications in real‐world use.^20^ This topic has received little attention to date.^21^ Based on national registries in Sweden, the aim of the current study was to analyse the use of biologics for psoriasis treatment in real‐world practice, with a particular focus on dose adjustments of individual biologics and their cost impact.

## Methods

### Study design

**Figure 1:**
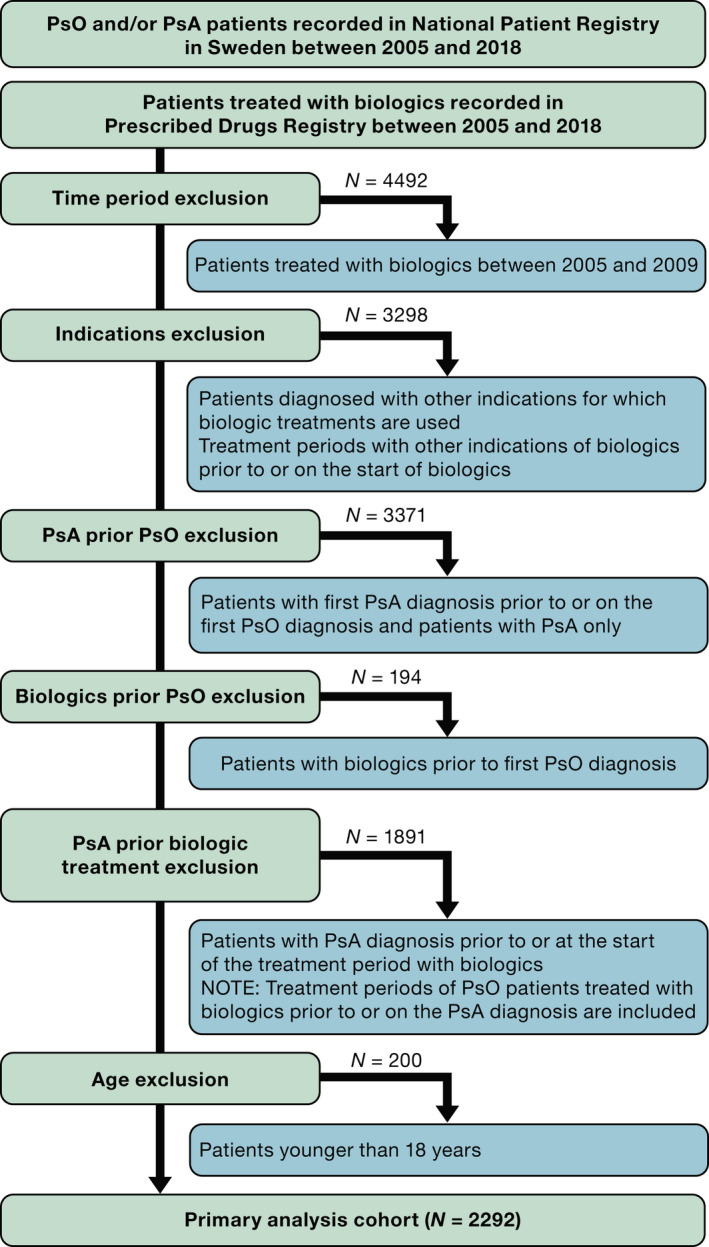
STROBE (strengthening the reporting of observational studies in epidemiology) diagram of the primary study population; PsA, psoriatic arthritis; PsO, psoriasis. [Colour figure can be viewed at wileyonlinelibrary.com]

This was a retrospective, observational, longitudinal cohort study of adults with moderate to severe psoriasis, using data from three Swedish national registries between 2010 and 2018 (Fig. 1); details of the study design have been reported previously.^22^ Patients with registered diagnoses (other than psoriasis) for which biologic treatments may be used and patients diagnosed with psoriatic arthritis (PsA) were excluded (Fig. 1). Eight biologics were included as previously reported,^22^ noting that in Sweden, anti‐TNF drugs are recommended as first‐line treatment, with physicians free to choose any other treatment thereafter. Only treatment groups that included ≥ 50 patients were used for the dosing analyses to ensure sufficient patient numbers for each of the drugs (adalimumab, etanercept, secukinumab, ustekinumab).

### Dosing of biologics

The recommended dosing scheme (Table 1) was available to clinicians in the Summary of Product Characteristics (SmPC) for each biologic on the European Medicines Agency website (https://www.ema.europa.eu/en). For most biologics, treatment involves initial induction at a higher dose, followed by a maintenance period. Following recommendations, the dosing of biologics is not adjusted according to body weight, with the exception of ustekinumab.

**Table 1:** Recommended dosing of biologics and mean treatment costs according to the summary of product characteristics for each drug.

Parameter	ADA^27^	ETA^28^	SEC^29^	UST^23^
Induction period	Start dose of 80 mg Week 0	–	300 mg Weeks 0, 1, 2, 3, 4	Start dose of 45 mg Week 0. Patients with body weight > 100 kg: 90 mg Week 0
Maintenance period	40 mg every other week, starting Week 1^b^	25 mg twice weekly or 50 mg once weekly^c^	300 mg monthly	45 mg every 12 weeks, starting Week 4. Patients with body weight > 100 kg: 90 mg every 12 weeks, starting Week 4
Mean cost per syringe/vial (SEK)^a^	714.12	1047.54	5837.13	33 546.11

ADA, adalimumab; ETA, etanercept; SEC, secukinumab; SEK, Swedish Kroner; UST, ustekinumab.

^a^ Mean costs of the least expensive available drug were calculated based on list prices extracted from www.apoteket.se in August 2020.

^b^ Dose can be increased to 40 mg once weekly or 80 mg alternate weekly at Week 16, depending on response.

^c^ Dose may be doubled to 50 mg twice weekly for up to 12 weeks if a response is not achieved.

### Statistical methods

Analyses were descriptive, as previously noted.^22^ Only the first treatment episode of a specific biologic was analysed for dosing. A treatment episode was defined as treatment with a specific biologic during which patients persisted with treatment, i.e. the gap between administrations (from the end of supply of the former administration to the administration date of the next) was < 90 days, in line with previous literature and supported by sensitivity analyses in this study. Biologic dosing patterns were analysed longitudinally.

Analyses included quantification of the accumulated syringes/vials at a given time point as recommended by SmPC guidance and proportion of treatment episodes with accumulated syringes/vials above, equal to and below those recommended by SmPC guidance. Because dispensations in real‐life practice do not occur exactly at the weeks defined by the guidelines, a formula was applied to calculate the corrected number of accumulated syringes/vials, as follows:

$$\text{Corrected accumulated syringes}/\text{vials}=\frac{\text{accumulated syringes}/\text{vials}}{\text{accumulated weeks}}\times \text{weeks}\;\mathrm{per}\;\text{guideline}$$

The numbers of syringes/vials used per day per dispensation were calculated from treatment start and from treatment end; the latter was defined as the final dispensation plus supplied days at the final dispensation, with no new dispensations of the same treatment within the next 90 days.

Real‐world costs incurred for each biologic were calculated by multiplying the mean number of accumulated syringes/vials for a given period by the list price per syringe/vial in Swedish Kroner (SEK; 10.4 SEK equivalent to 1 EUR, August 2020; costs extracted from www.apoteket.se in August 2020). To determine costs for each biologic, recorded at the anatomical therapeutic chemical level, costs for the least expensive available drug (originator or biosimilar) were used. These costs were then compared with the costs that would be expected if dosing had followed the guideline recommendations in Table 1. For direct cost comparison between biologics, the mean cost of dose increments was simulated for each biologic over a standard 24‐month period.

SAS version 9.4 software (SAS Institute Inc., Cary, NC, USA) was used for all data management and analysis.

## Results

### Baseline characteristics

In total, 178 347 patients diagnosed with psoriasis and/or PsA between 2005 and 2018 were identified in the National Patient Register. Of these, 15 738 had at least one biologic administration described in the Prescribed Drug Register for 2010–2018. Following exclusions, including removing patients with PsA, the primary analysis cohort comprised 2292 patients (Fig. 1).

Patient baseline characteristics are presented overall and for each biologic in Table 2. Overall, the mean ± SD time from first observable visit to specialty care of psoriasis was 7.7 ± 3.7 years and the time from psoriasis diagnosis to biologic initiation was 5.6 ± 3.6 years. Median persistence for biologics overall was 23.8 months (95% CI 21.6–26.2), ranging from 16.3 months (95% CI 4.5–19.0) for etanercept to 49.3 months (95% CI 38.0–59.1) for ustekinumab.

**Table 2:** Baseline characteristics of patients overall and in each cohort treated with a biologic for psoriasis by number of treatment episodes.

Parameter	Overall^a^	ADA	ETA	SEC	UST	IXE
Number of treatment episodes, *n*	3747	1448	1125	441	564	51
Number of patients with ≥ 1 treatment episode, *n*	2292	1046	974	394	488	50
Male, *n* (%)	2230 (59.5)	946 (65.3)	675 (60)	267 (60.5)	354 (62.8)	31 (60.8)
Age at diagnosis, mean (SD)	42.1 (14.2)	41.1 (13.9)	42.6 (14.4)	42.6 (14.0)	43.3 (14.4)	39.8 (14.4)
PsA diagnosis after index treatment, *n* (%)	287 (7.7)	115 (7.9)	110 (9.8)	12 (2.7)	34 (6.0)	0 (0)
PsO‐related concomitant medication, *n* (%)						
Overall	2950 (78.7)	1123 (77.6)	933 (82.9)	344 (78.0)	446 (79.1)	42 (82.4)
Topical calcipotriol	2067 (55.2)	646 (44.6)	547 (48.6)	185 (42.0)	256 (45.4)	23 (45.1)
Topical steroids	1726 (46.1)	752 (51.9)	610 (54.2)	257 (58.3)	341 (60.5)	26 (51.0)
Systemic treatments^b^	817 (21.8)	342 (23.7)	294 (26.1)	64 (14.5)	73 (12.9)	4 (7.8)
Previous biologic treatments, *n* (%)^c^						
0	2292 (61.2)	862 (59.5)	904 (80.4)	202 (45.8)	254 (45.0)	6 (11.8)
1	874 (23.3)	274 (18.9)	121 (10.8)	179 (40.6)	224 (39.7)	38 (74.5)
2	341 (9.1)	163 (11.3)	60 (5.3)	53 (12.2)	53 (9.4)	5 (9.8)
3	136 (3.6)	78 (5.4)	25 (2.2)	6 (1.4)	21 (3.7)	2 (3.9)
≥ 4	104 (2.8)	71 (4.9)	15 (1.3)	1 (0.2)	12 (2.3)	0 (0)
Duration of PsO (from the first PsO diagnosis to end of the follow‐up), years						
Mean (SD)	7.7 (3.7)	7.9 (3.7)	7.0 (4.5)	7.8 (3.8)	8.5 (3.5)	9.1 (3.5)
Median (Q1, Q3)	7.4 (4.7, 10.8)	7.9 (4.9, 11.0)	6.7 (4.0, 10.0)	7.4 (5.0, 10.2)	8.5 (5.7, 11.4)	9.3 (5.9, 13.0)
Range	0.1–14.6	0.2–14.0	0.1–14.0	0.7–14.3	0.4–14.6	1.0–14.1
Hospitalization for PsO^c^ 1 year prior to initiation of biologic, *n* (%)	104 (2.8)	36 (2.5)	33 (2.9)	14 (3.2)	20 (3.6)	0 (0)
Outpatient visits for PsO^d^ 1 year prior to initiation of biologic, *n* (%)	2885 (77.0)	1157 (79.9)	860 (76.4)	329 (74.6)	468 (83.0)	37 (72.6)

ADA, adalimumab; ETA, etanercept; IXE, ixekizumab; PsA, psoriatic arthritis; PsO, psoriasis; Q1, quartile 1; Q3, quartile 3; SEC, secukinumab; UST, ustekinumab.

^a^ Patients could use several different biologic treatments throughout their treatment course; data are not shown for biologics with *n* < 50 (48 patients were treated with golimumab, 23 were treated with certolizumab pegol, 12 were treated with guselkumab, 11 were treated with infliximab and 4 were treated with brodalumab); however, they are included in the overall group.

^b^ Systemic treatments were defined as methotrexate, apremilast and immunosuppressive therapies.

^c^ Patients who were treated with biologic treatments before 2010 were excluded. Patients might have had > 1 treatment episode with biologics. If the patient had 2 treatment episodes with biologics, the second treatment episode was regarded as biologic‐experienced and therefore marked as having 1 previous biologic treatment episode.

^d^ PsO as primary diagnosis.

### Dosing analysis

Mean (weighted) doses were above recommended levels for all biologics investigated; for adalimumab, etanercept, secukinumab and ustekinumab these were 13%, 23%, 8% and 3% higher, respectively, which was at 264 weeks (5.1 years), 268 weeks (5.1 years), 139.1 weeks (2.7 years) and 352 weeks (6.7 years), respectively.

**Figure 2:**
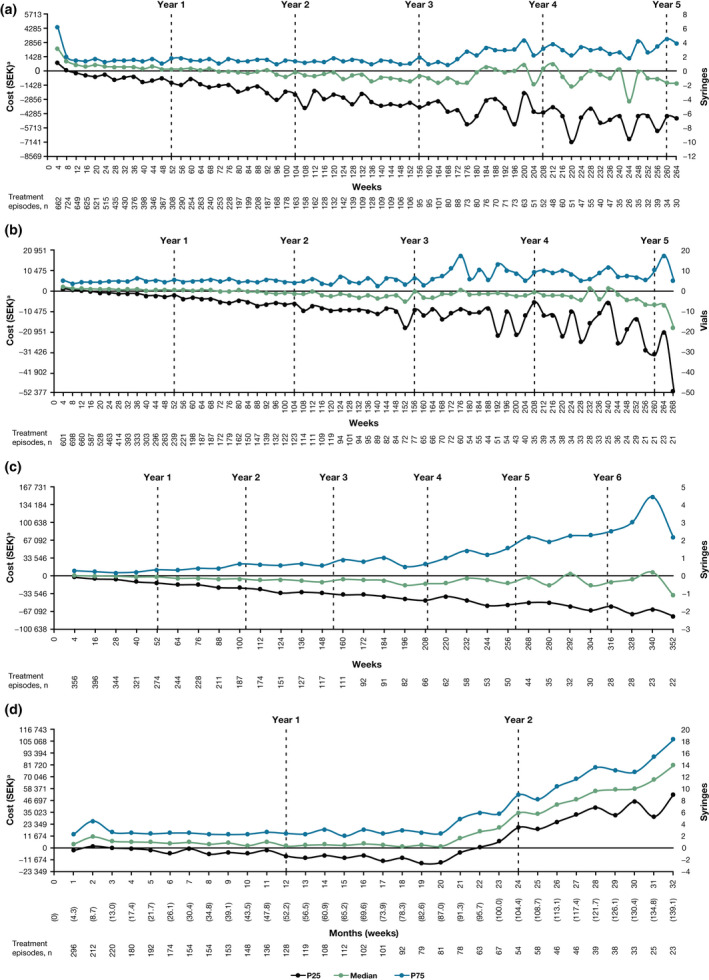
(a–d) Dosing analysis for each of the biologic treatment groups that included ≥ 50 patients; accumulated syringes (vials for etanercept) vs. syringes (vials for etanercept) that should be accumulated per the summary of product characteristics: (a) adalimumab; (b) etanercept; (c) ustekinumab; (d) secukinumab. P25, 25th percentile; P75, 75th percentile. ^a^Costs were calculated based on mean cost per syringe (Table 1). SEK, Swedish Kroner. [Colour figure can be viewed at wileyonlinelibrary.com]

Longitudinal analysis showed that the median dosing was approximately the amount recommended in the SmPCs for adalimumab (within ± 2 syringes during most time points; Fig. 2a), etanercept (within ± 5 vials; Fig. 2b) and ustekinumab (within ± 1 syringe during most time points; Fig. 2c) throughout the treatment period, although there was a decrease near the end of this period for patients receiving etanercept. For secukinumab, there was an increase in the median number of accumulated syringes above SmPC recommendations at the start of treatment, which coincided with the induction and early maintenance period for this biologic; the median number of accumulated syringes remained relatively stable up to 87 weeks of treatment, at which point the median number of accumulated syringes steadily increased to the end of the treatment period (Fig. 2d). Longitudinal analysis of ustekinumab revealed high variance around the median (Fig. 2c); a proportion of patients (in the 75th percentile) showed a steady increase in accumulated syringes after approximately 52 weeks (around the first year of treatment) until the end of the treatment period (at Week 352).

#### Cost impact of dose adjustments

The cumulative costs compared with the expected costs (calculated based on SmPC recommendations; Table 1) reflected the changes in dosing in the longitudinal analysis (Fig. 2).

The calculation of simulated, cumulative costs of drug increments over a 24‐month period revealed that the cost of dose increments was lowest for adalimumab (4740 SEK), followed by ustekinumab (9560 SEK), secukinumab (23 348 SEK) and etanercept (23 842 SEK).

## Discussion

This retrospective, observational registry study in Sweden in patients with moderate to severe psoriasis revealed that for adalimumab, etanercept, secukinumab and ustekinumab in treatment groups with at least 50 patients each, the mean (weighted) doses were 3–23% higher than SmPC recommendations over the treatment period. As expected, the cumulative costs reflected the changes in dosing for each of the biologics. Longitudinal analyses showed that the pattern of dosing varied over time for individual biologics, with variability between the 25th and 75th percentiles. The observed increase in secukinumab dosing might be attributable to gradual loss of efficacy over time, as reported in previous studies.^11^, ^18^ For many patients, physicians may have preferred to escalate secukinumab dosing rather than switch treatment.

The recommended ustekinumab dosage is four syringes per year, consequently, a change of ± 1 syringe is equivalent to a 25% change in dose, which contributed to the large variability around the median between patients. Unlike other biologics in this study, dosing of ustekinumab can be adjusted according to body weight; however, data on weight were not available in the national patient registries, therefore assessment of dose modifications based on patient weight could not be determined. The recommended ustekinumab dosing is 45 mg every 12 weeks in patients who weigh ≤ 100 kg, and 90 mg every 12 weeks in those who weigh > 100 kg.^23^ In our study, approximately 20% of patients initiated on 45 mg were escalated to the 90 mg dose, presumably because of perceived suboptimal efficacy.

The weighted mean dose for etanercept was relatively stable throughout, with some variability from approximately the third year of treatment. It is noteworthy that, while remaining within SmPC recommendations, the etanercept dose could be increased from 25 mg twice weekly or 50 mg once weekly to 50 mg twice weekly for up to 12 weeks if response was not achieved. The influence of this change in dose on overall dosing could not be ascertained from our registry data. Low patient numbers up to the fifth year might also have influenced the reliability of the dosing pattern observed.

Our findings are broadly consistent with the limited evidence available from previous real‐world studies, which reported that biologics are frequently prescribed in practice at doses that are higher than recommended by the manufacturer.^11^, ^15^ In studies reporting specific biologics, dose elevation occurred in a higher proportion of patients with etanercept compared with other biologics, including adalimumab and ustekinumab.^21^, ^24^, ^25^ Our study, although not directly comparable to others in terms of patient characteristics, doses and treatment durations, supports these findings.

Although analyses of registry data cannot provide insights into reasons for biologic dose increases in individual patients, perception of suboptimal efficacy is likely to be a major contributor. Notably, etanercept showed the greatest mean dose increase in the current study. In addition to suboptimal efficacy, other factors, such as lack of tolerability and market factors, including the availability of new biologics, contribute to changes in the prescription of individual biologics. Additional research is warranted to identify the impact of these factors in clinical practice. Future research could also study why physicians increase dosing rather than switch treatments. There may be a perception that, through increasing the dosing, patients will be able to stay on that specific treatment for longer, even though switching to more effective treatment options might be a better strategy. Additional studies could investigate persistence and patient outcomes when comparing doses of biologics that are at, above and below the recommended doses.

Analyses of costs associated with dose changes over time are important for understanding the economic consequences of using individual biologics. Dose escalation can have an impact on the cost and cost‐effectiveness of treatments, which vary according to the cost of the individual biologics (Table 1).^26^ We found that the accumulated costs in the secukinumab group were notably higher than expected for the median dose, reflecting increased dosing over time. Costs were higher than predicted in the 75th percentile of the etanercept and ustekinumab groups, but these were offset by lower costs in the 25th percentiles. We assessed only the economic impact of dose changes over time based on actual drug costs. However, treatment costs might also include costs resulting from management of AEs in patients who are overdosed.^21^, ^24^ Therefore, the cost implications associated with dose changes are likely to be greater.

The impact of real‐world dosing was calculated by assessing the difference in cumulative costs for real‐world doses minus those for guideline‐recommended doses, which were simulated over 24 months for each individual patient. This cost analysis showed that adalimumab had the lowest cost of dose increments and etanercept the highest. This reflects the substantial impact on cumulative cost of both the mean cost per syringe/vial and the dosing frequency. As these were per‐patient costs, a further calculation including population size and drug‐duration distribution would be required to determine the societal costs of the dose increments.

An important strength of this study is that it used national registry data from Sweden, including detailed data on prescriptions linked to diagnoses at the individual‐patient level; selection bias is removed because the datasets cover the entire population and include all prescriptions dispensed. Furthermore, the study was longitudinal and covered a greater time period than would be possible with a prospective study. However, there are also a few limitations that are inherent in the design of retrospective, observational studies. Data were not collected specifically for the purposes of this study, so treatment groups were not matched for patient characteristics and reasons for dosing adjustments were not assessed. For this analysis, several assumptions were made, although their accuracy could not be verified based on the data collected; these included the specific timing of when and if a drug was used in relation to a prescription being dispensed. It was also assumed that the day of dispensation was the date of use of the treatment. Finally, this study used descriptive statistics without statistical evaluation of the differences between biologics. Further research is warranted to confirm our findings and explore the reasons for dose increases, and further characterize their clinical and economic impact.

## Conclusions

Our study demonstrates that dose adjustments over time are common for biologics in clinical practice. The overall increases in dose to levels above recommendations for all the biologics analysed have economic consequences with relevance to the cost and cost‐effectiveness of the individual therapies.

**What's already known about this topic?**

- Dose adjustments of biologics are frequent in clinical practice, but few studies have analysed the dose adjustments of individual biologics in the real‐world treatment of moderate to severe psoriasis.

**What does this study add?**

- This patient‐level registry study from Sweden shows that the average dosing of biologics is above guideline recommendations, with a trend to increased dosing over time.
- This increase in dosing might indicate that current biologic therapies are frequently perceived to be suboptimal.

## Conflict of interest

AE has received research funding from Pfizer, Eli Lilly, Novartis, AbbVie, Janssen Pharmaceuticals, the Danish National Psoriasis Foundation, the Simon Spies Foundation and the Kgl. Hofbundtmager Aage Bang Foundation; and has received honoraria as a consultant and/or speaker from AbbVie, Almirall, LEO Pharma, Samsung Bioepis Co. Ltd, Pfizer, SUN Pharmaceuticals, Galapagos NV, Eli Lilly, Novartis, Galderma, Dermavant, UCB, Mylan, Bristol Myers Squibb and Janssen Pharmaceuticals. JF and MNS are employees of Parexel International. EA is a current employee, and RK and JBH are former employees of LEO Pharma A/S. LÅL has received consulting fees from LEO Pharma, Biogen, Novartis, AbbVie, Galderma, UCB and Pfizer.

## Funding

This study, with the exception of the contributions of AE and LÅL, was funded by LEO Pharma A/S, which also participated in the interpretation of data and review and approval of the presentation, and funded editorial assistance.

## Ethics statement

The study was designed, implemented and reported in accordance with the Guidelines for Good Pharmacoepidemiology Practice of the International Society for Pharmacoepidemiology, the STROBE (Strengthening the Reporting of Observational Studies in Epidemiology) guidelines and the ethical principles specified in the Declaration of Helsinki. Ethics approval for this study was provided by the independent ethics committee in Stockholm, Etikprövningsnämnderna. The study was approved by the Regional Stockholm Ethics Committee (reference number 2018/1:3). Informed consent not applicable.

## Data Availability

The datasets generated during and/or analysed during the current study are available from the corresponding author on reasonable request.
